# Simulated consultations: a sociolinguistic perspective

**DOI:** 10.1186/s12909-016-0535-2

**Published:** 2016-01-15

**Authors:** Sarah Atkins, Celia Roberts, Kamila Hawthorne, Trisha Greenhalgh

**Affiliations:** Centre for Research in Applied Linguistics, Trent Building, University of Nottingham, Nottingham, NG7 2RD UK; Department of Education & Professional Studies, King’s College London, Franklin-Wilkins Building, Waterloo Road, London, SE1 9NH UK; Duke of Kent Building, Faculty of Health and Medical Sciences, University of Surrey, Surrey, GU2 7XH UK; Nuffield Department of Primary Care Health Sciences, University of Oxford, Oxford, OX2 6GG UK

**Keywords:** Simulated consultations, OSCE, Communication skills, Interpersonal skills, Assessment, Diversity, Sociolinguistics

## Abstract

**Background:**

Assessment of consulting skills using simulated patients is widespread in medical education. Most research into such assessment is sited in a statistical paradigm that focuses on psychometric properties or replicability of such tests. Equally important, but less researched, is the question of how far consultations with simulated patients reflect real clinical encounters – for which sociolinguistics, defined as the study of language in its socio-cultural context, provides a helpful analytic lens.

**Discussion:**

In this debate article, we draw on a detailed empirical study of assessed role-plays, involving sociolinguistic analysis of talk in OSCE interactions. We consider critically the evidence for the simulated consultation (a) as a proxy for the real; (b) as performance; (c) as a context for assessing talk; and (d) as potentially disadvantaging candidates trained overseas. Talk is always a performance in context, especially in professional situations (such as the consultation) and institutional ones (the assessment of professional skills and competence). Candidates who can handle the social and linguistic complexities of the artificial context of assessed role-plays score highly – yet what is being assessed is not real professional communication, but the ability to voice a credible appearance of such communication.

**Summary:**

Fidelity may not be the primary objective of simulation for medical *training*, where it enables the practising of skills. However the linguistic problems and differences that arise from interacting in artificial settings are of considerable importance in *assessment*, where we must be sure that the exam construct adequately embodies the skills expected for real-life practice. The reproducibility of assessed simulations should not be confused with their validity. Sociolinguistic analysis of simulations in various professional contexts has identified evidence for the gap between real interactions and assessed role-plays. The contextual conditions of the simulated consultation both expect and reward a particular interactional style. Whilst simulation undoubtedly has a place in formative learning for professional communication, the simulated consultation may distort assessment of professional communication These sociolinguistic findings contribute to the on-going critique of simulations in high-stakes assessments and indicate that further research, which steps outside psychometric approaches, is necessary.

## Background

This paper addresses issues arising from the use of simulated patients in assessments of clinical consulting, in particular the linguistic difficulties of interacting in such settings and how far they reflect a practitioner’s real consulting abilities. Simulated patients are lay people or professional actors trained to portray a patient with a particular condition in a standardised way. As well as their use in practice and training for medical practitioners, they play an important role in formal assessment, such as the objective structured clinical examination (OSCE) for undergraduates [1, 2] and licensure examinations for postgraduates [3–5]. One advantage of this assessment format is that it helps ensure everyone has a standardised, equitable and repeatable experience [6–8]. However, given that such exams can demonstrate significant differences in pass rates between demographic groups, such as, the Membership of the Royal College of Physicians ‘Practical Assessment of Clinical Examination Skills’ (PACES) exams and Membership of the Royal College of General Practitioners’ Clinical Skills Assessment (CSA) [9–11], the construct validity of simulations is an important research question. Recreating a linguistically authentic medical interaction may not be the primary objective of simulation when used for medical *training*, where it enables practice and focussing in on particular skills, particularly technical skills. Recent research evidence has also shown the important uses of simulation in communication skills training for medical teams, not least because of the facility it provides practitioners to record and reflect on how an interaction has unfolded [12]. However, the linguistic differences and difficulties of simulation are of considerable importance in *assessment*. In many high-stakes summative exams, the simulated consultation is not used to enable the medical practitioner to reflect on and develop their own communication skills, but rather for an external party to measure a candidate’s competence and assign a grade. When using the simulated consultation for assessment, particularly where interpersonal and communication skills are being marked, we must be sure that the exam construct and the linguistic requirements placed on candidates adequately embody the skills expected for real-life practice.

The authenticity of the interaction is particularly pertinent when assessing communication skills. The clinical consultation is not a technical procedure, but an emotionally charged interpersonal interaction of high social significance and linguistic complexity [13]. When considering the appropriateness of simulation in assessing this complexity, we need a different kind of research - sited in a humanistic rather than psychometric paradigm. Sociolinguistic research provides a useful means of interrogating and debating these issues. Sociolinguistics is a field which systematically studies the way language and society inter-relate. It looks at how people use language in their everyday lives and how language-in-context creates the complex social world. The tools of sociolinguistic research are real recordings of spoken language, to examine evidence of how different contexts and social backgrounds affect the talk we produce and how it is evaluated by others. This focus on evidence from actual interactions is an important one here. Prior research on simulated consultations has largely addressed psychometric properties of particular tests and scenarios such as internal consistency (e.g. using Cronbach’s alpha), generalisability, inter-rater reliability, predictability (e.g. of subsequent examination success), discriminatory power (ability to distinguish consistently between ‘good’ and ‘poor’ examinees), as well as protocols and procedures for quality control [3, 14–18]. A crucial question remains as to how far such scenarios reflect real consulting abilities. Both simulated patients and those being assessed, when asked after the event, tend to rate their experience as ‘realistic’ [19, 20]. Various studies have also found that unannounced simulated patients, trained to present with a particular scenario in a kind of ‘mystery shopper’ approach, went undetected by medical practitioners [6, 21]. Yet there remain crucial differences between real and simulated consultations, particularly as they are used in assessment, that cannot easily be evidenced by the mystery shopper method or participants’ retrospective accounts.

Sociolinguistic analysis can unpick the subtly different interactional discrepancies that a simulation produces. It looks at direct evidence from the interactions produced in situ, rather than relying on asking participants to reflect back on an interaction. Sociolinguistics often employs techniques of ‘discourse analysis’ to identify important features and fine-grained characteristics of talk, such as grammatical structure, turn-taking between speakers, intonation and the integration of non-verbal communication [22]. These can systematically evidence the characteristic linguistic features that occur in a particular setting - and how the talk is created by, as well as constitutive of, the social relationships in that context. Sociolinguistics is therefore interested in the choices that speakers make when they use language and what those choices and variations might mean for the evaluation of speakers (page 16) [23].

Integrated with this theoretical approach in sociolinguistics, is a fundamental interest in how language produces power relations in society. Professional discourses in particular, which largely consist of goal-oriented encounters, often demonstrate a degree of power-imbalance [24]. We can evidence these power relationships in the ‘microphysics’ and fine-grained detail of our everyday practices [25], including our professional talk. There are asymmetrical relationships in terms of who is expected to speak at certain points, who should show politeness and which speakers are meant to demonstrate their domain-specific, professional knowledge [26]. Power relations in the setting of an exam role-play differ from real-life clinical encounter, since the role-player has a very different position to that of a patient and there is also a more powerful third participant in the examiner, observing the interaction. Given the different relationships of participants, interactional differences in simulated consultations compared to real-life are perhaps to be expected. The environment in assessed simulations is (intentionally) decontextualized and scenarios involving invasive physical examinations [5] and a range of patient groups such as those with multi-morbidity, limited English or a very different communicative style [27] are often (though not invariably) excluded. Yet candidates are asked to behave *as if* these scenarios were real [28, 29].

This sociolinguistic approach to language and professional communication was recently used in a 3-year study of the Royal College of General Practitioners’ Clinical Skills Assessment (CSA) [27, 30], which we draw upon in this paper. As well as this we draw on analytic work around final-year undergraduate medical OSCEs by Roberts et al. [31]. De La Croix and Skelton on undergraduate OSCEs [32–34], Seale et al.’s linguistic study of OSCE examinations [28], Niements on the use of role-play in training [35], and O’Grady and Candlin on the Royal Australian College of General Practitioners' licensing exam [36]. Some linguistic evidence on the use of simulation for assessing professional communication outside the medical field is also drawn upon, particularly Stokoe’s [37, 38] account of the linguistic patterns in simulated police interviews. Evaluating this evidence collectively helps us flesh out this debate paper with a fuller picture of the complexities of using simulation. Although simulated consultations and OSCE exams do vary in their setup, some of the essential commonalities, like the semi-scripted standardised part of role-players, the timed cases and marking descriptors, render our sociolinguistic discussion relevant to this wide genre of assessment. The paper addresses the following four themes, identified from the literature review and our own research, in considering the simulated consultation: (a) as a proxy for the real; (b) as performance; (c) as a context for assessing talk and (d) as potentially disadvantaging candidates trained overseas.

## Discussion

### The simulated consultation as a proxy for the real

Social interaction cannot ultimately be standardised. While there are some relatively stable, overarching features of a consultation, such as the phases to be performed and the general history to be conveyed by the patient, at the minute level of turn-by-turn talk standardisation becomes difficult and small differences in delivery are inevitable. There is a tension, then, between the degree of standardisation of any scenario (hence, its replicability) and its reflection of the real (its authenticity), since 100 % standardisation would require the simulated patient to reproduce a script robotically. In reality, while the simulated patient plays a character and helps depict a contextual hinterland in answer to candidates’ questions, he or she must draw on their own interactional resources to manage the interaction itself [28].

To understand how simulations are experienced differently from real consultations, we must ask, “what ‘maintenance work’ needs to be done by both parties to maintain the semblance of reality?”. To do this, we draw on the work of sociologist Erving Goffman, whose seminal essay ‘Frame Analysis’ addressed the question, “under what circumstances do we think things are real?” [39]. Goffman argued that the sense of feeling an activity is real depends upon our sense of self as we relate to others. Each interaction creates and reinforces a shared reality to keep the relationship going [40]. We attend to others, become involved in the to and fro of talk, however momentarily, since we have what Goffman calls a moral requirement to display ourselves in ways that others expect of us.

His concept of ‘frame’ describes this socially defined reality. In any given stage of an encounter, speakers and listeners establish or negotiate what is going on: we are in the frame of a passing conversation, a preliminary chat about the weather before the consultation proper begins, an examination and so on. The frame constitutes what is happening and also works as a filtering process through which general principles of conduct apply. For example, when a doctor tells a patient that chances of recovery are high, both sides can understand that they are in a ‘reassurance’ frame within this shared moment of reality. Different frames can be invoked, and indeed evidenced, through changes in linguistic behaviour by the participants. For example, in a case from our CSA research [27], a simulated patient presents with menorrhagia. The candidate indicates that he wants to do a "quick abdominal examination", in the frame of a routine element of the diagnosis. But when he responds to the simulated patient’s query by saying “we look for any abnormal growth”, the simulated patient becomes alarmed. The candidate then shifts the frame from information giving to one of reassurance and self-correction.

At any time in an encounter, Goffman argues (page 156–200) [39], we can experience multiple frames. For example, in an OSCE-style exam, the frame of showing empathy to a role-playing patient is nested in a frame of displaying competence to an examiner, which in turn is nested in the institutional frame of the overall assessment process. For this reason, the values associated with empathy are not seriously committed to or felt as real because they are anchored in a more fundamental frame, related to simulated performance in the exam. While role-player and trainee/candidate can put on a surface performance that is realistic, the assessor must decide whether the candidate is demonstrating ‘real caring’. This makes any simulated consultation a hybrid activity in which real qualities (subjectively experienced) are assessed through the unreal, requiring a considerable amount of interactional work to sustain the talk and illusion of a real consultation [28].

Goffman calls an activity that does not fit within the frame of the moment a ‘frame-break’. For example, candidates in simulated consultations often do not know whether they are expected to carry out a physical examination ‘for real’. They may commence a physical examination frame, only to be interrupted by the examiner either verbally or by handing them a card with key physical findings. Candidates must then rapidly shift frame to the preliminaries of diagnosis. We found such shifts were typically marked by disfluencies and/or hesitations, even with highly successful candidates, as the candidate worked to maintain the simulated case and ignore any interaction with the examiner (page 53) [27]. To justify a simulated consultation as a proxy for the real obscures its limitations and complexities, many of which only become apparent when analysing their interactional detail. It is in this linguistic detail of simulations that we can really identify the different communicative competences that come to the fore in simulated consultations, which may not be the competences required for real-life practice.

### The simulated consultation as performance

One of the concerns voiced about OSCE examinations is that they test acting skills as much as they do professional communication [30, 41]. Niements describes how role-played interactions "cannot reproduce the orientations of real interactions...[W]hat is authentic to those *users* when they “live” a specific situation cannot be authentic to *trainers/trainees* when they play it" [42] (p. 317). A number of studies have addressed the types of ‘acted’ behaviour such settings consequently produce, what de la Croix and Skelton have called "the language game of role-play" [32]. Seale et al. explore how different ‘frames’, real and fictitious, are invoked through talk in simulations [28]. They find subtle moments in which attention is drawn to the fictitious nature of role-play, citing an example of humorous comments made about an entirely invented paediatric patient, that both the candidate and the role-player are pretending is present (page 183). In their analysis, using the fictitious nature of role-play to create humour is a means for the candidate to achieve rapport with the actor, not rapport with a ‘patient’. So there are multiple roles and identities at play in simulations and these can be evidenced in the communication. In answering the question on ‘authenticity’, Seale et al. ultimately do suggest that experience of participants in a role-play is fundamentally different from that of a real-life interaction and that the candidate must do much more interactional ‘work’ to keep the illusion up (page 181).

Of course, real consultations also require some level of performance, but to properly understand the differences we must unpack what ‘acting’ and ‘performing’ mean in these interactional situations. To do so, we can draw on Goffman’s depiction of life as drama – i.e. we present ourselves on the world as a stage, ‘performing’ in different ways to different ‘audiences’ in different settings (everyday, professional, institutional and so on) [43]. We perform all the time in the everyday, managing impressions of ourselves in what Goffman called ‘facework’ [44].

Goffman distinguished the banal and intimate performances of the everyday that occur ‘backstage’ from professional behaviour, which is largely ‘front-stage’ [43] – a term he used to refer to activities like the waiter at table, the doctor in the surgery or the teacher in class. Here there are constraints on behaviour in terms of manner, quality of attention and emotions, and the performance has an ‘audience’ that evaluate the competence displayed [45, 46]. Importantly, understanding professional behaviour as a performance does not undercut its values. For example, to care for a patient may involve masking frustration or fatigue in order to care better. When institutions require this professional behaviour to be monitored and assessed, however, it becomes an institutional performance. Evaluation of professional performance becomes institutionalised as observers rate and record performance and implement rewards and sanctions. There is a heightened awareness of the need, on the part of the professional, to perform expressively, a “heightened mimicry” [28] and, on the part of the assessor, “a license … to regard the act of expression and the performer with special intensity” (page 11) [46]. However, it is important to make the distinction between a heightened performance for institutional purposes (e.g. someone pointedly looking in the mirror when taking a driving test) and a simulated performance (someone pretending to look in the mirror).

In simulation, the environment is mutually constructed as an unreal activity. In her analysis of emotions in theatre acting, Konijin discusses the way actors must monitor how far the emotions they are acting out accord with the inner model of what the play should convey [47]. The actor’s task is not to convey sincere emotions but to play out words and actions that convince the audience of the authenticity of their character within the terms of the drama. At the same time they monitor their own experience of acting and so experience a ‘dual consciousness’. In a simulation, likewise, the trainee or candidate has to work hard to create a synthetic reality – one that convinces the audience/observer, but not one that is real to candidates in terms of consequences for patients: an institutionalised display rather than a professional investment, all the while monitoring their conduct vis-à-vis the examiner. In sum, simulation is a multi-layered performance for both role-player and candidate requiring some of the skills of an actor.

### The simulated consultation as a context for assessing talk

The design of OSCE-style exams bring five other complexities, relating to the quality of talk to the candidate’s task, adding burdens and reducing the ‘real’. We consider: (i) the talk-heavy nature of the consultations; (ii) the design and timing of cases; (iii) the shift of power to the role-player; (iv) standardised scenarios but individual emotional responses and (v) who fails such assessments – and why? We establish these themes from the authors’ study of the CSA [27] and from an overview of the linguistic research on simulations [28, 32–37], but draw on these findings to debate the particular implications for *assessment*.

#### The talk-heavy nature of the consultations

In simulated consultations, it is primarily talk-in-interaction that is assessed. To succeed in simulated scenarios, candidates must work harder or ‘over perform’, holding a higher proportion of the conversational floor (between 67–77 %) than in everyday consultations [34]. Research by Seale et al. identifies the complex, additional linguistic work required from candidates in simulations [28] and research on the Royal Australian College of General Practitioners’ licensing examination identified how role-played scenarios require a complex, hybrid discourse from the GP candidate [36]. Collectively, these findings suggest that simulated consultations require actions and skills to be verbalised by the candidate to a much greater degree than in everyday clinical work.

Talk in simulated assessments is also relatively decontextualized, without the shaping role of the computer [27, 48] or any of the other props and interruptions of real consultations. Decontextualised environments incur more talk [49] and, in an environment such as this, lead to talk becoming intensely focussed on. In addition, there is no continuity of care, so shared, unspoken knowledge between doctor and patient can play no part. This potentially diminishes the types of relationships and interactions that can be experienced by doctor and patient in the simulated consultation. Relationship building over time and the deep values inherent in building professional capability [50, 51] are overshadowed by an externally timed case where surface skills must be made explicit (e.g. enacted or voiced) for assessment. This simultaneous amplification and reduction is most apparent in the interpersonal domains of assessment, as we discuss below.

#### The design and timing of cases

The design of cases for simulated consultations moves the focus from the how of patient care to the why of the particular selected case. Both students and candidates are primed to fear the trip-wire that comes with the case: “learners sometimes think there are hidden aspects…they are being asked to discover, akin to peeling away the skins of an onion until the flesh is found” (page 67) [52].

In a high-stakes examination, this ‘Sherlock Holmes’ factor can mar or make success [27]. It turns the candidate into a timekeeper, dealing with concerns superficially so that the putative puzzle of the case can be resolved. They may stop in mid-sentence when the whistle blows or pack in questions or information as the last minute ticks by. The strictly timed structure for simulated consultations produces very different openings and closings from that identified in real consultations. For example, in real clinical encounters, doctors raising new topics at the likely end of the encounter is rare but, conversely, closings are often extended conversational exchanges which build the doctor-patient relationship more generally [53].

#### The shift of power to the role-player

Sociolinguistic research has identified how asymmetrical interactions, where one speaker has more power than another, show small-scale differences in talk. Medical consultations are necessarily asymmetrical. The movement in recent years towards patient-centredness and shared decision making has not fundamentally altered this, since asymmetry stems at least partly from the doctor’s knowledge [54]. But in simulated consultations, candidates must manage the fact that “the power relation is inverted, because knowledge and judgment rest with the simulated patient rather than with the physician student” (page 266) [55]. De la Croix and Skelton identify a higher number of interruptions from role-players across 100 third-year OSCE exams, suggesting a position of greater interactional power compared to findings on the linguistic behaviour of real-life patients [32–34]. Not only do the simulated patients know the case and how it should play out [34], but in examiner feedback sessions for research on simulations [27], examiners noted that the simulated patients positioned themselves in an actorly manner. They put demands on candidates that patients usually would not and showed familiarity with the exigencies of the case through their language [27].

Evidence from outside medical education has also shown the shift in power relations between speakers when interacting in simulations. Stokoe conducts a nuanced account of the conversational inauthenticities of role-play for police interviews [37], particularly the more elaborate and sometimes humorous way in which conversational actions are performed in these false settings, where the stakes for participants are entirely different from those where a real defendant is being interviewed. There is linguistic evidence, then, for how participants must orient themselves in acted, simulated settings, monitoring their performance and conducting extra linguistic work to maintain the illusion of a real interaction.

#### Standardised scenarios but individual emotional responses

The role-player’s power is made more complex by the shift from the institutional persona of the actor/patient to the instinctive resources of the private person. In other words, the role-player works with a hybrid of acting behaviour and their own, individual interactional resources. While careful training is used to standardise ‘patients’, the role-player is usually not working to a tightly scripted part. He or she is given guidance to react to the candidate in a natural way, to fulfil interactional criteria (Table 1). If the candidate’s performance is unclear or irritating to the role-player, then the role-player can respond in accord with their inner emotions (the irritation feels real, even though the setting is simulated).

**Table 1:** Examples of instructions to role-player from the CSA: Behaviour/Demeanour/Body language

‘Case 1’
Reticent, trying to appear unconcerned.
A bit resentful if the doctor appears to be telling you off.
‘Case 2’
Try to build a good rapport with doctor and don’t keep information back.
You are familiar with GPs and hospitals, so you are comfortable with the doctor.

All parties in fact, must draw on their own interactional resources to make sense of the encounter. Even where a middle class actor acts a convincingly troubled and inarticulate teenager, they cannot gainsay their own interpretive processes (e.g. they can mumble or remain silent but they cannot *not* understand). Examiners not only have to judge this hybrid of simulation and instinctive resources, they also have to manage their own mix of instinctive reactions to how others interact, their own professional expectations and the formal categories of the examination. As one said, “A lot of the time, I am comparing them to me and what I’m used to” [27]. This mix of habits of talk, interpretation and evaluation (on the one hand) and standardised judgements (on the other) are most problematic in the domain of interpersonal skills, where subjective interpretation is necessary to interpret what counts as ‘rapport’ or ‘sensitivity’. This became particularly evident in feedback sessions with examiners, as the following section explores.

#### Who fails such assessments – and why?

We have noted the heavy focus on talk in simulated consultations. Communication or interpersonal skills are often explicitly assessed with their own marking criteria in medical OSCEs, but can also become implicitly judged across all other domains, since professional actions like data-gathering and clinical management must also be performed through effective communication [27]. The metric of reliability also tends to reinforce the unspoken assumption that there is an implicit ‘best way’ of scoring highly in the interpersonal domain. Candidates in simulated consultations routinely produce formulaic phrases such as "Can you tell me a bit more about…" "I understand how you feel" or "I’m sorry to hear about that", with a greater frequency and often in different sequential positions, than is found in real life practice [27]. Some of these mimic the phrases recommended in communication skills textbooks and their extensive use in simulations may be inevitable in an environment in which talk is being observed and assessed. This is a finding corroborated by Roberts et al. [31] in a study of undergraduate medical OSCEs, where the use of elicitation phrases such as "How do you feel about that?" could be interpreted as sounding overly trained if used in the wrong location (page 8–9). In an essay on the experience of being a role-player evaluating candidates in US medical exams, Jamison points out that to gain marks, empathy and compassion must be ‘voiced’ and that (perhaps as a consequence) candidates seemed either aggressively formulaic in their insistence, "that must be really hard", or saturated with humility "Would you mind if I – listened to your heart?" (page 4–5) [56]. There have been similar findings on simulations in professional settings outside medicine, such as Stokoe’s research on police interview role-plays, in which communication directives from training manuals are overtly used in the openings, in a way which they are not in real-life police interviews, potentially for the benefit of a marker [38].

It seems to be a consequence of the assessed, simulated setting then, that participants use these formulaic, trained professional phrases and interactional moves with a much higher frequency than real-life. In exams such as the CSA, high scoring candidates also produce 32 % more of these exam-modelled utterances than weaker candidates. Yet these phrases appear much less frequently in real consultations [27]. Interestingly, weaker CSA candidates who also produce these types of phrases, albeit slightly less frequently, were assessed as formulaic in examiner feedback:

It seems just very formulaic and a lot of it seems learned. ‘I understand why you would be worried’, ‘What kind of thought went through your mind when you made this appointment’ which kind of is an attempt to do the right thing but to me it just felt very crass… [27]

Detailed analysis of stronger candidates’ talk showed that they knew how to play the game: they customised formulaic phrases so they sounded more real and sincere, adding in little hesitations, colloquialisms and changes in intonation (page 59–61) [27]. In such circumstances, the ‘empathy telling’, already a simulation of feeling and perception, has to be further worked on to invoke a convincing suspension of disbelief: a double simulation or to extend Konijin’s concept of ‘dual consciousness’ [47], a ‘triple consciousness’, consisting of the candidate’s own sense of themselves as professionals, the consciousness that they must simulate a professional encounter – and in addition within the institutional frame – must work on the formulaic phrases of the simulation so that they sound more sincere to examiners. It is in these small details of talk, here in the small variations in delivery of exam-modelled phrases, that we can see how power and social relationships are constituted in the micropractices of interaction and its evaluation [25].

In terms of construct validity, does the simulated consultation measure what it purports: the interpersonal capabilities expected of a doctor? The answer is a complex one. Though the simulation may be good at testing skills such as giving explanations and structuring the consultation, there are a number of linguistic features which do not mimic real-life practice. For example, it examines competence in using additional communicative resources to make exam-induced ‘voiced’ phrases sound sincere and to manage the triple consciousness required to perform to examiners. In terms of assessment theory, there is a "construct-irrelevant variance" [57] in which certain know-how is assessed which is not a requirement of real consultations.

### Simulated consultations as potentially disadvantaging candidates trained overseas

While all assessments may require ‘exam skills’ to some degree, when one group of candidates fares much worse than another, as occurs in many of these assessments [27], the fairness of these exam-constructed requirements needs to be carefully considered. There is wide recognition that for many candidates trained outside their home country for the assessment, simulations are often a new phenomenon and that, like any type of assessment, lack of familiarity affects performance [58]. The simple solution offered is that this group need more practice with simulations. However, detailed sociolinguistic analysis suggests that simulations may cause difficulties for this group of candidates in other ways as well.

As indicated above, simulations lead to more talk, more formulaic phrases and more work to ensure that such talk sounds sincere. This focus on talk and how it sounds in contexts of intense assessment puts particular pressure on those whose style of communicating is different from the majority of examiners and also, perhaps, the patient role-players. Small differences in such subtle features as intonation, word stress and other small markers of speech can be amplified and read off as showing negative characteristics, such as formulaic responses or not engaging, attracting lower marks in the interpersonal (page 32–73) [27]. Additionally, since it can be difficult to make standardised, simulated cases reflect the same variation as real-life consulting, performance will not reflect the ability to interact effectively and flexibly with a diverse patient population. In many such exams, while UK graduates are not assessed on consulting in linguistically challenging situations, International Medical Graduates, many of whom consult regularly in another expert language within the British multi-cultural context, have no opportunity to display this skill as they might use it in their everyday practice. Such competence in linguistically and culturally challenging situations is increasingly important for medical practitioners treating diverse patient populations, both in the UK and globally. It is perhaps the biggest challenge for assessing medical practitioners’ interpersonal competence in our modern-day context of globalised, mobile and diverse societies.

## Summary

A review of sociolinguistic approaches to simulations demonstrates that simulated assessment, even when it is ‘realistic’, shows some crucial differences to the communicative competences found in real-life practice. Talk is always a performance in context and in simulations, the role-playing patient, the candidate and the examiner all have to work hard to maintain the illusion. Candidates who can handle the social and linguistic complexity of this somewhat artificial, standardised situation score highly – yet what is being assessed is not real communication but the ability to voice a credible appearance of such communication. It follows that if communication skills are assessed purely through simulated patients, this may not reflect the real consulting abilities of candidates. We must question whether simulations replace the values–led development of medical students with ‘playing the game’ of simulation [50, 51, 59]. The ability of doctors to form enduring therapeutic relationships with patients may not be adequately reflected in the “colonisation [of medicine] by the technologies of the unreal” [60].

The discipline of sociolinguistics offers an evidenced approach to these questions around professional communication. In this paper, we have introduced three core sociolinguistic concepts relevant to the assessment of communication in medicine: that the particular variety of talk in simulated consultations separates it out from the talk in real consultations; that the notion of ‘frame’ is used to understand how we relate to and make our talk real to each other and that this reality breaks down in institutionally assessed communication; and that micro-features of talk feed constantly into our evaluation of others and, in high-stakes assessments, can have large consequences on the trajectory of an interaction. While a single awkward moment is unlikely to lead to failure, in settings of intense evaluation, perceived infelicities such as an unfilled pause or formulaic phrase become amplified. The cumulative effect of such micro-features may lead to a candidate being judged as "not developing rapport" or as showing inadequate responsiveness to "verbal and non-verbal cues" and an overall negative impression of interpersonal abilities.

Although a number of studies have identified that simulated interactions show important differences from real-life professional communication [27, 28, 33–37], we are not arguing that simulation has no place in teaching or assessment. Much of medical practice consists of skills that are more or less technical in nature and which can be both taught and assessed effectively using simulated patients (the rationale behind the ‘skills lab’) [61]. Formative simulated consultations have great value in the safety they afford learners to make and learn from mistakes, as well as to ‘slow down’ the consultation to study what has happened. Summative simulated assessments, however, must carefully consider the difficulties of assessing interpersonal skills in this setting. Hence, we do not seek to bury the OSCE, but in introducing the sociolinguistic perspective, we do seek to debate its level of validity for assessing communicative and interactional aspects of clinical performance. Furthermore, we believe the evidence identified in a number sociolinguistic studies of simulated interaction [27, 30, 58] requires us to consider carefully what we mean by ‘fairness’ in assessment and how we might better assess communication skills in settings of cultural and linguistic diversity.

## Abbreviations

CSA: clinical skills assessment
MRCGP: Examinations for ‘Membership of the Royal College of General Practitioners’
OSCE: objective structured clinical examination
RCGP: Royal College of General Practitioners
